# Coagulation Factor XIII Subunit A Is a Biomarker for Curative Effects and Prognosis in Malignant Solid Tumors, Especially Non-small Cell Lung Cancer

**DOI:** 10.3389/fonc.2021.719085

**Published:** 2021-12-15

**Authors:** Yujiao Luo, Bin Li, Ji Li, Yang Zhang, Mingyang Deng, Chunhong Hu, Wenzhe Yan, Zhiguang Zhou, Guangsen Zhang

**Affiliations:** ^1^ Department of Hematology, Section of Hemostasis and Thrombosis, Institute of Molecular Hematology, The Second XiangYa Hospital, Central South University, Changsha, China; ^2^ National Clinical Research Center for Geriatric Disorders, Department of Geriatrics, Xiangya Hospital, Central South University, Changsha, China; ^3^ Department of Oncology, The Second XiangYa Hospital, Central South University, Changsha, China; ^4^ Department of Metabolism and Endocrinology, The Second XiangYa Hospital, Central South University, Changsha, China

**Keywords:** biomarker, non-small cell lung cancer, prognosis, curative effect, tumor-associated macrophages (TAMs), factor XIII subunit A, factor XIII/transglutaminases

## Abstract

**Background:**

The expression of coagulant factor XIII subunit A (FXIII-A) is significantly increased in some types of cancer cells and tumor-associated macrophages (TAMs). However, few studies on plasma FXIII-A in cancer patients have been conducted and have shown contradictory results, so the relationship of plasma FXIII-A with the progression and prognosis of malignant tumors is still unknown. This study explored the association of plasma FXIII-A with a curative effect and the prognosis of patients with malignant solid tumors.

**Methods:**

We monitored plasma FXIII-A before and during systemic therapy and assessed its relationship with the curative effect and prognosis of malignant solid tumors, especially non-small cell lung carcinoma (NSCLC), by propensity-adjusted, multivariable logistic regression analysis and survival curve, in a prospective study of 1147 patients with different types of malignant solid tumors. The influencing factors of plasma FXIII-A were also analyzed.

**Results:**

We found that D-dimer (D2) = 1 mg/L was the inflection point for the association between FXIII-A and D2: FXIII-A was significantly negatively correlated with D2 (r = -0.39, *p <* 0.01) and FDP (r = -0.40, *p* < 0.01) in D2 > 1 mg/L but uncorrelated with D2 or FDP in D2 ≤ 1 mg/L, which provided a method to find a more realistic plasma FXIII-A level. Plasma FXIII-A was positively correlated with age, platelets, lymphocytes, monocytes and carcinoembryonic antigen (CEA). It was found for the first time that plasma FXIII-A was abnormally significantly increased (FXIII-A > 150%) in post-therapy patients, especially in NSCLC and lung metastasis patients, and the incidence of FXIII-A > 150% in lung adenocarcinoma was 16 times higher than that in lung squamous carcinoma. FXIII-A > 150% proved to be an independent risk factor for disease progression in NSCLC patients (OR=5.74, 95% CI: 1.20-27.60, p = 0.029), predicting poor efficacy. The marked decrease in plasma FXIII-A (FXIII-A < 40%) was related to coagulation disorders and poor prognosis with a short survival time (median survival time of 4 months).

**Conclusions:**

Plasma FXIII-A has the potential to be a real-time biomarker with bidirectional indicator effects to assess curative effects and prognosis in malignant solid tumors, especially NSCLC.

## Introduction

Coagulant factor XIII (FXIII) circulates in plasma in a tetrameric form (FXIII-A_2_B_2_), consisting of two potentially active catalytic A subunits (FXIII-A_2_) and two carrier B subunits (FXIII-B_2_). It stops bleeding by cross-linking adjacent fibrin chains to form insoluble clots in the last step of the clotting cascade, and a very small amount of plasma FXIII (5%-10%) is required for normal blood clotting. It has long been considered a simple coagulation factor; however, FXIII is a transglutaminase. In addition to its role in hemostasis, it also plays an important role in neoplasms (1, 2).

FXIII‐A is the functional subunit of FXIII that is expressed mostly in cells of bone marrow origin, such as platelets, megakaryocytes, monocytes, and macrophages. In recent years, the expression of FXIII-A was found to be significantly upregulated in some types of cancer cells and tumor-associated macrophages (TAMs). The expression of FXIII-A was increased in acute myelomonocytic leukemia, acute monocytic leukemia and acute promyelocytic leukemia, suggesting that FXIII-A might be used as a biomarker for differentiating myeloblastic and monoblastic leukemias (3, 4) and for prognostic risk stratification (5–7). Kiss, F et al. reported that normal lymphoid precursors and mature lymphocytes do not contain FXIII-A, but the expression of FXIII-A in B cell acute lymphoblastic leukemia cells is significantly increased (8). In addition to leukemias, abnormal changes in FXIII-A have also been observed in malignant solid tumors (9), such as non-small cell lung carcinoma (NSCLC) (10), sebaceous neoplasms (11) and colorectal cancer (12). Moreover, the transcription level of FXIII-A increased 2.5-fold in monocytes in the spleen of a mouse model of lung squamous carcinoma (LUSC) (13). Fibrin deposition and a number of tumor-associated macrophages (TAMs) expressing FXIII‐A are located within and around neoplastic tissues (14). FXIII‐A plays an important role in the growth and metastasis of malignant tumors (15, 16).

However, some studies on plasma FXIII-A in cancer patients have shown contradictory results; for example, patients with acute leukemia, neuroblastoma, non-Hodgkin’s lymphoma, alveolar rhabdomyosarcoma and other solid tumors were found to exhibit secondary FXIII-A deficiency and bleeding manifestations (17, 18). There are many reasons for the abnormal changes of plasma FXIII-A levels in cancer patients, but two important areas need to be distinguished: the first is the change of hemostasis in the presence of malignant tumors, and the other is related to the function of FXIII-A transglutaminase. One of the main aims and findings of the present study is to clarify the background of the changes in plasma FXIII-A levels.

Moreover, existing studies on FXIII-A have mostly focused on hematological malignancies and intracellular FXIII-A expression, while few studies have assessed plasma FXIII-A and malignant solid tumors. In addition, most studies had single sample types, small sample sizes, single data collection time points and other confounding factors. The relationship of plasma FXIII-A with the curative effects and prognosis of malignant solid tumors is still unknown. This study explored the association of plasma FXIII-A with curative effects and prognosis, as well as the influencing factors of the plasma FXIII-A in patients with malignant solid tumors. We monitored plasma FXIII-A and coagulation parameters before and during systemic therapy and assessed the relationship of plasma FXIII-A with the curative effects and prognosis of malignant solid tumors, especially NSCLC, in a prospective study of 1147 patients with different types of malignant solid tumors. We also analyzed the influence of age, sex, pathological type, pathological stage, history of tumor surgery, treatment and changes in coagulation or hemogram on plasma FXIII-A levels.

## Materials and Methods

### Study Subjects

A total of 1147 inpatients with malignant solid tumors who were admitted to the Department of Oncology, The Second Xiangya Hospital, Central South University, between November 2018 and November 2019 were enrolled randomly in the prospective study. The study protocol was approved by the local ethics committee. The follow-up time of the present analysis was 6 months. Eighty-seven age- and sex-matched healthy controls were also included in the study (males: 59, females: 28; age: 52.9 ± 13.8 years). All the healthy controls had no history of hemorrhagic disease, cardiovascular disease, cancer or anticoagulants, and their coagulation parameters were normal.

### Blood Sampling and Plasma Preparation

Peripheral venous blood (2.7 mL) was drawn at enrollment prior to the start of each systemic therapy into 4 mL vacutainer tubes containing 0.109 M citrate (9:1 vol/vol); plasma was isolated by centrifugation at 3000 rpm for 10 minutes. All samples were anonymized and tested in a blinded manner at the Laboratory of Hemostasis and Thrombosis, the Second Xiangya Hospital, Central South University.

### FXIII-A Level and Coagulant Parameters Assay

Freshly isolated plasma samples were assayed immediately for FXIII-A antigen and the coagulant index, including D-dimer (D2), prothrombin time (PT), activated partial thromboplastin time (APTT), fibrinogen, and fibrin/fibrinogen degradation products (FDP). The tests were performed on an ACL-TOP 700 automatic coagulometer (HemosILTM, IL, MA, USA) and completed within 4 h of collection. FXIII-A detection was carried out using the Factor XIII Antigen assay kit (HemosILTM, IL, MA, USA) and immunoturbidimetry. The concentration of FXIII-A antigen is expressed as a percentage. The reference range of plasma FXIII-A at the Laboratory of Hemostasis and Thrombosis, the Second Xiangya Hospital, Central South University, is 70%-150%. The therapeutic interval for each course was approximately 1 month. The course of treatment was set as n(t), and the number of plasma FXIII-A detection times was set as n(m).

### Collection of Relevant Data

Hemogram parameters (leukocytes, erythrocytes, hemoglobin, platelets, lymphocytes, and monocytes; Sysmex-XN20, Japan) and carcinoembryonic antigen (CEA; Hybiome-AE240, China) tested simultaneously with plasma FXIII-A, as well as information on the history of tumor surgery and therapeutic methods were collected. The medical records and imaging data of patients were obtained from the hospital medical records platform. Follow-up was achieved directly using a telephone questionnaire.

### Statistical Methods and Data Analysis

All statistical analyses were performed with SPSS version 25.0 (IBM) and GraphPad Prism version 8.2.1 (GraphPad software). The Kolmogorov-Smirnov test was used for normality analysis. Clinical characteristics were compared between groups using Student’s t-test or Mann–Whitney U test for continuous measures and χ^2^ test for categorical variables. The Spearman test was used to analyze the correlation of plasma FXIII-A with coagulation parameters, hemogram parameters and CEA. A curve fitting the scatter plots of D2 and FXIII-A was generated to determine the inflection point. The survival curve of patients with FXIII-A < 40% was generated after a follow-up period of 6 months. We focused on analyzing the changes in plasma FXIII-A levels in NSCLC patients with different pathological types or stages by Student’s t-test or Mann–Whitney U test and the influencing factors for abnormal increases in plasma FXIII-A (FXIII-A > 150%) in NSCLC patients by logistic regression. NSCLC patients were divided into two groups: the FXIII-A > 150% group and the FXIII-A ≤ 150% group, and 1:1 propensity score matching (PSM) was used to match the age, sex, pathological types and pathological stages of the two groups to exclude confounding factors. The treatment response to treatment based on the RECIST criteria evaluation showed progressive disease (PD), stable disease (SD), partial response (PR) and complete remission (CR). The association of FXIII-A > 150% with NSCLC PD was analyzed by propensity-adjusted, multivariable logistic regression analysis. The comparison groups were all age- and sex-matched. A *P* value < 0.05 was considered statistically significant by a two-sided test. Continuous variables are expressed as the mean ± standard deviation, and categorical variables are expressed as the number (percentage).

## Results

### An Abnormal Increase in Plasma FXIII-A Occurred in Post-Therapy Patients, Especially NSCLC Patients

**Figure 1:**
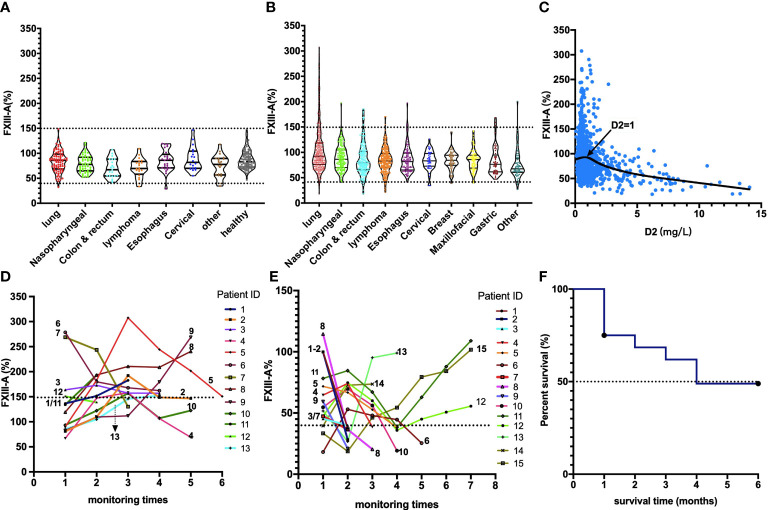
Distribution, association and monitoring of the plasma FXIII-A levels in cancer patients. **(A)** Plasma FXIII-A levels in pre-therapy patients with malignant solid tumors; **(B)** Plasma FXIII-A levels in post-therapy patients with malignant solid tumors; FXIII-A > 150% was found only in the post-therapy patients, the incidence of FXIII-A > 150% in lung cancer is the highest, the incidence of FXIII-A < 40% was not specific for neoplasm types and occurred both in pre- and post-therapy. **(C)** The scatter graph with trend line based on plasma FXIII-A level and D2 level in patients with malignant solid tumors; D2 = 1 mg/L is the inflection point for the correlation between FXIII-A and D2. **(D)** The monitoring of plasma FXIII-A in 13 NSCLC patients with PD. A persistent increase in plasma FXIII-A in all the patients, the plasma FXIII-A level in 9 patients (patient ID: 1-9) lasted over 150% for more than two test times (two courses of treatment). **(E)** The monitoring of plasma FXIII-A levels in the patients with FXIII-A < 40%. Ten patients ((patient ID: 1-10) who died within four months had a marked decrease in plasma FXIII-A, and with an ending FXIII-A < 40%. The plasma FXIII-A of the surviving patients (patient ID: 11-15) all bottomed out and rose again. **(F)** Survival curve of the patients with FXIII-A < 40%, median survival time is four months.

**Figure 2:**
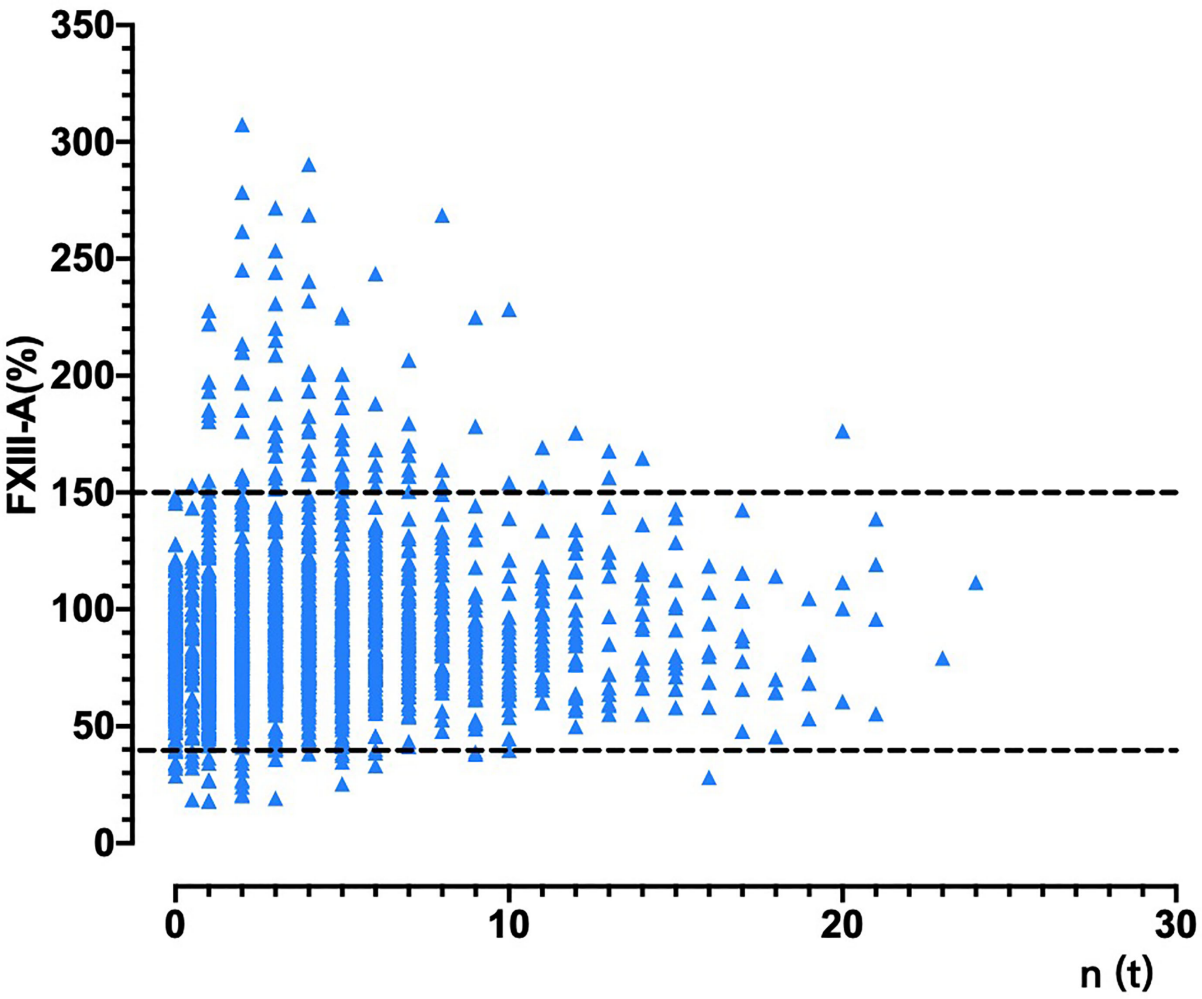
The changes in plasma FXIII-A levels in patients with malignant solid tumors during treatment. The abscissa is the corresponding times of treatments n(t) at each test of plasma FXIII-A in patients with malignant solid tumor. For patients with malignant solid tumors that had been treated in another hospital, the times of tests before the first treatment in our hospital was identified as 0.5 times. FXIII-A > 150% was found only in the post-therapy patients, FXIII-A < 40% occurred both in pre- and post-therapy patients.

The neoplasm classification and distribution of the plasma FXIII-A level in the 1147 patients included in this study are presented in **Table 1** (each type of tumor with a sample size greater than 20 was grouped independently). The plasma FXIII-A level in all pre-therapy patients was significantly lower than that in healthy controls (n = 247, 80.21 ± 21.01% vs. n = 87, 87.68 ± 18.86%, *p =* 0.007). The plasma FXIII-A levels in pre-therapy patients with nasopharyngeal carcinoma (*p =* 0.011), colon and rectal cancer (*p <* 0.001) and lymphoma (*p* = 0.001) were significantly lower than those in healthy controls, but all were in the normal range. In view of the range of plasma FXIII-A healthy controls (46.8% - 143.8%) and the reference range of plasma FXIII-A (70% - 150%), we chose 40% and 150% as the cutoff points to perform the stratification analysis. Notably, the abnormally significant increase in plasma FXIII-A (FXIII-A > 150%) occurred only in post-therapy patients with malignant solid tumors and was most common in lung cancer patients, especially NSCLC patients. There were 64 patients with plasma FXIII-A > 150%: 43 lung cancer patients all had NSCLC, and 21 patients had other styles of malignant solid tumors. Moreover, 13 of 21 (61.9%, including colon & rectum cancer, gastric cancer, biliary tract carcinoma, nasopharyngeal carcinoma, lymphoma and melanoma) patients with other types of malignant solid tumors had lung metastasis. However, the incidence of FXIII-A < 40% was not specific for neoplasm types and occurred both pre- and post-therapy (**Figures 1A, B** and **2**).

**Table 1:** Descriptive analysis of plasma FXIII-A in study objects.

Cancer	All patients	Pre-therapy	FXIII-A > 150%	FXIII-A < 40%
	N = 1147	N = 247	N = 64(%)	N = 32
Lung	365	*83 (83.73 ± 20.08%)	43 (11.8)	7
Colon & rectum	155	*21 (69.91 ± 19.04%)	8 (5.16)	4
Nasopharyngeal	132	*53 (78.97 ± 17.00%)	1 (0.76)	
lymphoma	104	*21 (70.76 ± 19.02%)	3 (2.89)	7
Esophagus	74	*26 (84.80 ± 21.43%)	3 (4.05)	1
Cervical	56	*19 (88.93 ± 24.22%)		2
Breast	55	3		1
Maxillofacial	55	6		1
Gastric	35	4	2 (5.71)	2
Other	116	11	4	7
Gender				
Male	733	176	46	20
Female	414	71	18	12
Age(year)	54.6 ± 12.2	55.7 ± 12.2	58.9 ± 8.8	51.1 ± 15.6

Data shown as mean ± standard deviation or number (%). *Number (plasma FXIII-A level).

The linear regression analysis showed that plasma FXIII-A (Y) was significantly positively correlated with age (X) in both pre-therapy patients (Y=0.336*X+61.422, *p =* 0.002) and healthy controls (Y=0.350*X+68.963, *p =* 0.017). Therefore, age must be matched when conducting follow-up studies of plasma FXIII-A.

### The Association Between Hemogram Parameters, Coagulation Parameters and Plasma FXIII-A

Spearman analysis showed that the plasma FXIII-A level in patients with malignant solid tumors was significantly negatively correlated with D2 (*p* < 0.001), FDP (*p* < 0.001) and PT (*p* < 0.001) and positively correlated with fibrinogen (*p* < 0.001) (**Table 2**), suggesting that the decrease in FXIII-A might be related to increased fibrinolysis activity and enhanced coagulant consumption in patients. We also found that plasma FXIII-A levels were significantly positively correlated with the lymphocyte count (*p* < 0.001), monocyte count (*p* < 0.001) and platelet count (*p* = 0.007) (**Table 2**).

**Table 2:** Relativity analysis of hemogram parameters, coagulation parameters, CEA level and plasma FXIII-A level.

Variables	Total			D2 ≤ 1mg/L		
	n	r	P value	n	r	P value
D2	2009	**-0.15**	**<0.001**	1615	**0.02**	**0.324**
FDP	1838	**-0.15**	**<0.001**	1479	**0.03**	**0.217**
Fibrinogen	1875	**0.17**	**<0.001**	1509	**0.18**	**<0.001**
APTT	1867	-0.03	0.163	1504	**-0.08**	**0.003**
PT	1868	**-0.25**	**<0.001**	1505	**-0.2**	**<0.001**
Leukocyte	1941	-0.02	0.329	1575	0.02	0.362
Platelet	1941	**0.06**	**0.007**	1575	0.05	0.07
Lymphocyte	1940	**0.12**	**<0.001**	1575	**0.11**	**<0.001**
Monocyte	1940	**0.08**	**<0.001**	1575	**0.11**	**<0.001**
CEA	888	0.03	0.356	697	**0.11**	**0.003**

r, Spearman Rho coefficient.

#### D2 = 1 mg/L is the inflection point for the correlation between FXIII-A and D2, and plasma FXIII-A was positively correlated with CEA

According to the fitted curve, D2 = 1 mg/L was the inflection point for the correlation between FXIII-A and D2 (**Figure 1C**). Plasma FXIII-A was negatively correlated with D2 (n = 394, r = -0.39, *p <* 0.01) and FDP (n = 359, r = -0.40, *p* < 0.01) in D2 > 1 mg/L, but uncorrelated with D2 (n = 1615, *p =* 0.32) or FDP (n = 1479, *p =* 0.217) in D2 ≤ 1 mg/L. Since FXIII-A participates in fibrin cross-linking and D2 is the degraded product of cross-linked fibrin, the increase in D2 could indicate the accelerated consumption of plasma FXIII-A. FDP is the degraded product of cross-linked fibrin, fibrin and fibrinogen and displays a similar negative correlation with plasma FXIII-A, but theoretically, it is not as closely related as D2 and FXIII-A. D2 in patients were almost all within 1 mg/L when FXIII-A > 150%, but far more than 1 mg/L when FXIII-A < 40%. Moreover, all the other coagulation parameters were normal when FXIII-A > 150%, while obvious coagulation disorders were observed when FXIII-A < 40%; hemogram parameters were mostly within the normal reference range when FXIII-A > 150%, while erythrocyte count and hemoglobin concentration significantly decreased, and platelet count slightly decreased when FXIII-A < 40% (**Table 3**). The results confirmed that plasma FXIII-A is influenced by coagulation disorder in D2 > 1 mg/L but not in D2 ≤ 1 mg/L, and the basic level and associations of plasma FXIII-A in patients could be more truly reflected under the condition of D2 ≤ 1 mg/L.

**Table 3:** Analysis of coagulation parameters and hemogram parameters under the condition of plasma FXIII-A > 150% or FXIII-A < 40%.

Variables	FXIII > 150%			FXIII < 40%		
	n	IQR	Mean ± SD	n	IQR	Mean ± SD	Ref
D2 (mg/L)	99	0.45~0.76	0.71 ± 0.42	36	**1.60**~**7.68**	**5.07 ± 3.97**	0~0.50
FDP (mg/L)	87	2.50~4.17	3.63 ± 1.98	31	**8.72~33.11**	**24.48 ± 20.47**	0~5.00
Fib (g/L)	89	3.12~4.18	3.78 ± 0.95	32	**2.03**~3.89	2.94 ± 1.45	2.38~4.98
APTT (Sec)	88	28.9~32.7	31.2 ± 3.3	32	27.48~37.23	32.2 ± 6.3	25.0~38.0
PT (Sec)	88	10.5~11.5	11.1 ± 0.7	32	12.33~**14.75**	**14.9 ± 5.4**	8.0~13.0
FXIII-A(%)	100	158.1~209.7	188.6 ± 36.5	39	27.0~38.3	32.3 ± 6.8	70~150
W(10^9^/L)	94	4.35~7.19	5.92 ± 2.09	35	4.46~10.82	8.14 ± 5.67	3.50~9.50
R(10^9^/L)	94	3.69~4.42	4.05 ± 0.58	35	**2.65~3.98**	**3.03 ± 0.93**	4.30~5.80
Hb(10^9^/L)	94	113~135	123.0 ± 19.2	35	**81~118**	**97.9 ± 28.0**	130~175
P(10^9^/L)	94	163~265	222.7 ± 78.2	35	**84**~233	182.6 ± 144.5	125~350
L(10^9^/L)	94	1.18~1.89	1.56 ± 0.55	33	0.47~1.16	0.85 ± 0.58	1.10~3.20
M(10^9^/L)	94	0.34~0.56	0.47 ± 0.18	33	0.22~0.55	0.42 ± 0.30	0.10~0.60

IQR, inter quartile range; Ref, reference range; W, leukocyte; R, erythrocyte; P, platelet; Hb, hemoglobin; L, lymphocyte; M, monocyte.

Plasma FXIII-A was significantly positively correlated with the fibrinogen concentration and negatively correlated with APTT and PT in D2 ≤ 1 mg/L, consistent with the hemostatic function of FXIII-A. Interestingly, there was no significant difference in plasma FXIII-A between pre-therapy patients and healthy controls under the condition of D2 ≤ 1 mg/L (n = 207, 82.36 ± 19% vs. n =87, 87.68 ± 18.86%, *p =* 0.058), which was inconsistent with the overall sample analysis and suggested that the difference before treatment was affected mainly by the presence of a coagulation disorder.

Notably, plasma FXIII-A was significantly positively correlated not only with the lymphocyte count and monocyte count but also with the CEA level (r = 0.11, *p =* 0.003) in D2 ≤ 1 mg/L (**Table 2**). Plasma FXIII-A was not associated with leukocytes in either the overall sample analysis or D2 ≤ 1 mg/L.

### Patients With Advanced–Stage NSCLC Exhibited Higher Plasma FXIII-A Than Early-Stage NSCLC Patients

There were no significant differences in plasma FXIII-A between pre-therapy NSCLC patients (total: n = 72, FXIII-A: 85.30 ± 21.04%, *p =* 0.718; D2 ≤ 1 mg/L: n = 60, FXIII-A: 88.40 ± 18.89%, *p =* 0.768) and healthy controls (n = 87, FXIII-A: 87.46 ± 18.87%) or between pre-therapy NSCLC patients with adenocarcinoma and squamous cell carcinoma (total: n = 28, FXIII-A: 86.69 ± 22.81% vs. n = 39, FXIII-A: 85.48 ± 20.50%, *p =* 0.821; D2 ≤ 1 mg/L: n = 23, FXIII-A: 89.92 ± 19.16% vs. n = 33, FXIII-A: 88.02 ± 19.93%, *p =* 0.723) in the total sample or D2 ≤ 1 mg/L. The plasma FXIII-A in pre-therapy NSCLC patients with stage I + II disease was significantly lower than that of the patients with stage III + IV disease (n = 16, FXIII-A: 82.19% ± 10.00% vs n = 44, FXIII-A: 86.12 ± 23.46%, *p =* 0.040) when D2 ≤ 1 mg/L, consistent with the results of an existing study in which the FXIII-A activity of NSCLC patients in the advanced stage was higher than that of patients in the early stage (10). However, the difference was small, and plasma FXIII-A in both groups was within the normal reference range.

### Aging and Lung Adenocarcinoma Were the Influencing Factors of FXIII-A > 150% in NSCLC Patients

There were 324 NSCLC patients in the study, 50 patients who were missing or not monitored were excluded, and 274 available NSCLC patients were enrolled in the follow-up study. The 274 NSCLC patients were divided into two groups: the FXIII-A > 150% group (n = 43) and the FXIII-A ≤ 150% group (n = 231). The results showed significant differences in age (*p =* 0.044) and pathological type (*p <* 0.001) between the two groups, but there were no significant differences in sex, pathological stage, history of tumor operation or history of tumor operation within 1 year. Since all the patients with a history of tumor surgery in the FXIII-A > 150% group underwent surgery within 1 year and there was no significant difference in the history of tumor surgery between the two groups, we believed that it was more accurate to use the history of tumor surgery within 1 year to analyze the influence of the history of tumor surgery (tumor resection and wound repair of surgery) on plasma FXIII-A and subsequent treatment. Therefore, we included the history of tumor surgery within 1 year for analysis in subsequent studies. We included age, sex, pathological type, pathological stage and history of tumor surgery within 1 year in the multivariate logistic regression model and then performed stepwise binary logistic regression using the forward logistic regression method (forward LR) to include the variables in the model. Aging (OR = 1.05, 95% CI: 1.01-1.09, *p =* 0.017) and lung adenocarcinoma (OR = 5.33, 95% CI: 2.23-12.69, *p <* 0.001) were significant influencing factors of FXIII-A > 150% in NSCLC patients, and the incidence of FXIII-A > 150% in lung adenocarcinoma was significantly higher than that in LUSC.

**Figure 3:**
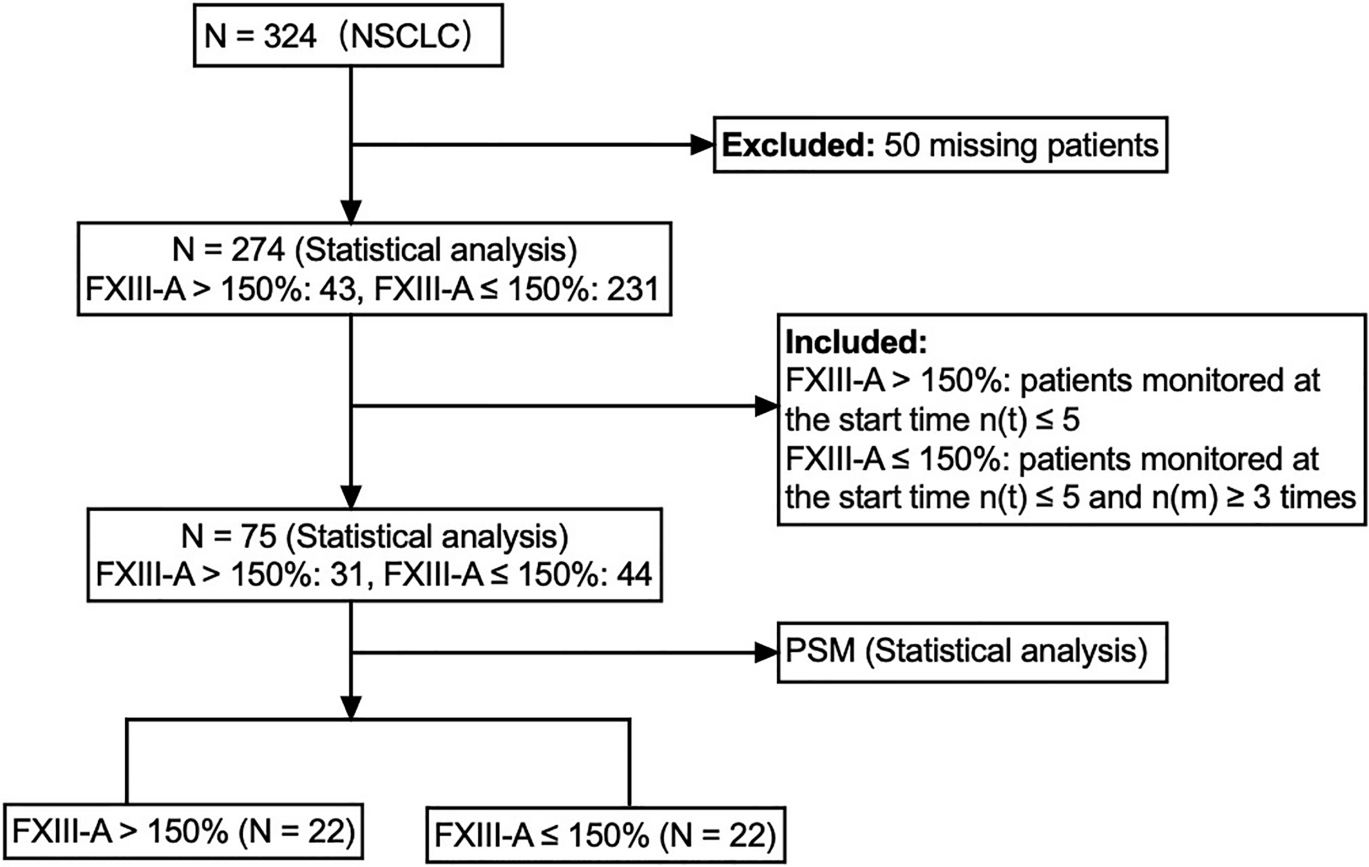
Flow chart of research steps.

To eliminate confounding factors, we further improved the inclusion criteria and included NSCLC patients who had the same monitoring period for subsequent analyses. We enrolled 23 NSCLC patients with FXIII-A > 150% who monitored plasma FXIII-A levels at the start time n(t) ≤ 3 to determine the median time point of occurrence of FXIII-A > 150%. The median time point occurred during the test at the fourth hospitalization (IQR: 3-5) based on the continuous FXIII-A monitoring data in the 23 NSCLC patients with FXIII-A > 150%. Then, we enrolled 31 patients with FXIII-A > 150% at the start time n(t) ≤ 5 as the study group and 44 patients with FXIII-A ≤ 150% at the start time n(t) ≤ 5 who were continuously monitored more than 3 times as the control group, to ensure that the two groups were in the same monitoring period (**Figure 3**). Given that all NSCLC patients received chemotherapy, we divided the therapeutic methods into combination therapy (chemotherapy+) and no combination therapy (chemotherapy alone). Since chemotherapy combined with targeted therapy or immunotherapy was the main therapeutic method in the combination therapy group, and only 2 NSCLC patients received chemotherapy combined with radiotherapy, the combination therapy group was also divided into a chemotherapy combined with targeted therapy group and a chemotherapy combined with immunotherapy group to explore the influence of treatment methods on plasma FXIII-A and curative effects. Univariate analysis showed differences in age and pathological type between the two groups (**Table 4**). We further analyzed associations between FXIII-A > 150% and related influencing factors (age, sex, pathological type, pathological stage, history of tumor surgery within 1 year, combination therapy, targeted therapy and immunotherapy) by multivariate logistic regression (forward LR). Aging (OR = 1.13, 95% CI: 1.03-1.23, *p =* 0.009) and lung adenocarcinoma (OR = 15.77, 95% CI: 3.77-65.95, *p <* 0.001) were still the independent influencing factors for FXIII-A > 150% in NSCLC patients, and the incidence of FXIII-A > 150% in lung adenocarcinoma was 16 times higher than that in LUSC. The abnormal increase in plasma FXIII-A in NSCLC patients was independent of sex, pathological stage, history of tumor surgery within 1 year, combination therapy, targeted therapy and immunotherapy. In addition, there were no differences in age (*p =* 0.772), pathological stage (*p =* 0.791), history of tumor surgery within 1 year (*p =* 0.680), combination therapy (*p =* 0.833), targeted therapy (*p =* 0.056) or immunotherapy (*p =* 0.595) between enrolled patients with lung adenocarcinoma and LUSC.

**Table 4:** Analysis of the FXIII-A > 150% group and the FXIII-A ≤ 150% group before and after PSM.

Characteristics		Before propensity matching			After propensity matching		
		FXIII-A>150%	FXIII-A≤ 150%	P Value	FXIII-A>150%	FXIII-A≤ 150%	P Value
		N = 31	N = 44		N = 22	N = 22	
Age (year)	61.7 ± 7.2		57.0 ± 7.3	0.011	60.0 ± 6.9	56.8 ± 7.1	0.165
Gender							
Male	25 (80.6)		31 (70.5)	0.910	17 (77.3)	13 (59.1)	0.195
Female	6 (19.4)		13 (29.5)		5 (22.7)	9 (40.9)	
Pathological type				<0.001			0.696
Adenocarcinoma	28 (90.3)		19 (43.2)		19 (86.4)	17 (77.3)	
Squamous carcinoma	3 (9.7)		25 (56.8)		3 (13.6)	5 (22.7)	
Pathological stage				0.527			0.342
I+II	6 (19.4)		5 (11.4)		4 (18.2)	1 (4.5)	
III+IV	25 (80.6)		39 (88.6)		18 (81.8)	21 (95.5)	
Surge (1year)				0.962			1
Yes	9 (29.0)		13 (29.5)		5 (22.7)	5 (22.7)	
No	22 (71.0)		31 (70.5)		17 (77.3)	17 (77.3)	
Combined therapy				0.802			0.210
Yes	16 (51.6)		25 (54.5)		12 (54.5)	16 (72.7)	
No	15 (48.4)		19 (45.5)		10 (45.5)	6 (27.3)	
Targeted therapy				0.565			0.195
Yes	6 (19.4)		11 (25.0)		5 (22.7)	9 (40.9)	
No	25 (80.6)		33 (75.0)		17 (77.3)	13 (59.1)	
Immunotherapy				0.587			0.75
Yes	11 (35.5)		13 (29.5)		8 (36.4)	7 (31.8)	
No	20 (64.5)		31 (70.5)		14 (63.6)	15 (68.2)	
Prognosis							
PD	13 (41.9)		6 (13.6)	0.006	10 (45.5)	3 (13.6)	0.021
SD	17 (54.8)		31 (70.5)	0.165	11 (50.0)	14 (63.7)	0.361
PR	1 (3.2)		7 (15.9)	0.170	1 (4.5)	5 (22.7)	0.188

Data shown as mean ± standard deviation or number (%). PD, progressive disease; SD, stable disease; PR, partial response.

### FXIII-A > 150% Was an Independent Risk Factor for Disease Progression in NSCLC Patients and Predicted Poor Efficacy

To analyze the association between FXIII-A > 150% and the curative effects of NSCLC patients, we followed the curative effect of the NSCLC patients by imaging for 4 months at 1-month intervals. The time point when FXIII-A > 150% first appeared was set as the starting point for follow-up in the study group; the second monitoring point was used as the starting point of follow-up in the control group (to ensure that the plasma FXIII-A levels were relatively stable and traceable over the monitoring period). Curative effects were divided into disease control (PR + SD) and progression (PD). 18 patients showed disease control (disease control rate, DCR 58.1%) and 13 patients (41.9%) progressed in the study group, while 38 patients showed disease control (86.4%) and 6 patients (13.6%) progressed in the control group during treatment. FXIII-A > 150% and lung adenocarcinoma were significantly associated with PD in NSCLC patients (*p* = 0.006, *p* = 0.005, **Tables 4** and **5**).

**Table 5:** Analysis of characteristics of patients with PD before and after PSM.

Characteristics	Before propensity matching			After propensity matching		
	PD	SD + PR	P value	PD	SD + PR	P value
	N = 19	N = 56		N = 13	N = 31	
Age (year)	58 ± 9.5	59 ± 6.8	0.551	58 ± 7.2	58 ± 6.9	0.944
Gender			1			0.652
Male	14 (73.7)	42 (75)		10 (76.9)	20 (64.5)	
Female	5 (26.3)	14 (25)		3 (23.1)	11 (35.5)	
Pathological type			0.005			0.459
Adenocarcinoma	17 (89.5)	30 (53.6)		12 (92.3)	24 (77.4)	
Squamous carcinoma	2 (10.5)	26 (46.4)		1 (7.7)	7 (22.6)	
Pathological stage			0.83			1
I+II	2 (10.5)	9 (16.1)		1 (7.7)	4 (12.9)	
III+IV	17 (89.5)	47 (83.9)		12 (92.3)	27 (87.1)	
FXIII-A			0.006			0.021
> 150%	13 (68.4)	18 (32.1)		10 (76.9)	12 (38.7)	
≤ 150%	6 (31.6)	38 (67.9)		3 (23.1)	19 (61.3)	
Surgery (1year)			0.133			1
Yes	3 (15.8)	19 (33.9)		3 (23.1)	7 (22.6)	
No	16 (84.2)	37 (66.1)		10 (76.9)	24 (77.4)	
Combined therapy			0.837			0.851
Yes	10 (52.6)	31 (55.4)		8 (61.5)	20 (64.5)	
No	9 (47.4)	25 (44.6)		5 (38.5)	11 (35.5)	
Targeted therapy			0.449			0.796
Yes	6 (31.6)	11 (19.6)		5 (38.5)	9 (29.0)	
No	13 (68.4)	45 (80.4)		8 (61.5)	22 (71.0)	
Immunotherapy			0.539			1
Yes	5 (26.3)	19 (33.9)		4 (30.8)	11 (35.5)	
No	14 (73.7)	37 (66.1)		9 (69.2)	20 (64.5)	

Data shown as mean ± standard deviation or number (%). PD, progressive disease; SD, stable disease; PR, partial response.

To further eliminate the confounding factors between the two groups and find a more real association between FXIII-A > 150% and curative effects, 1:1 PSM analysis was used to match the two groups based on age, sex, pathological type and pathological stage, resulting in 22 matched pairs in each group. FXIII-A > 150% was still associated with PD in NSCLC patients (*p* = 0.021) after PSM (**Table 5**).

In the multivariable logistic regression analysis before and after PSM adjusted for age and pathological type, FXIII-A > 150% remained significantly associated with poor efficacy and was proven to be an independent risk factor for disease progression (PD) in NSCLC patients. The risk of disease progression in NSCLC patients with FXIII-A > 150% was 6 times higher than that of NSCLC patients with FXIII-A ≤ 150% (OR=5.74, 95% CI: 1.20-27.60, p = 0.029) in the propensity-adjusted, multivariable logistic regression analysis (**Table 6**).

**Table 6:** Association of FXIII-A > 150% with PD in NSCLC.

	Before PSM			After PSM		
	OR	95%CI	P value	OR	95%CI	P value
Not adjusted	4.57	1.20-14.00	0.008	5.28	1.20-23.16	0.027
Adjusted for age	7.20	2.00-25.94	0.003	6.08	1.28-28.81	0.023
Plus pathological type	4.31	1.08-17.21	0.039	5.74	1.20-27.60	0.029

We also observed a persistent increase in plasma FXIII-A in the 13 NSCLC patients with PD in the study group, suggesting that the rapid progression of NSCLC was accompanied by the continuous expression and release of FXIII-A (**Figure 1D**).

### FXIII-A < 40% Was Related to Serious Complications, Poor Prognosis and Short Survival

Thirty-two patients with FXIII-A < 40% were followed up for 6 months. The results showed that the survival time was significantly shortened in patients with FXIII-A < 40%, with a median survival time of only 4 months; 8 patients (25%) died within 1 month (**Figure 1F**); moreover, 15 patients had serious cavity effusion (46.9%), 10 patients had bleeding or thrombosis (31.3%) and all patients had significantly abnormal coagulation parameters in plasma FXIII-A < 40% (**Table 3**). We observed the changes in the plasma FXIII-A of 15 patients monitored more than 2 times. All the patients who died had a marked decrease in plasma FXIII-A, with an ending FXIII-A < 40%; however, the plasma FXIII-A of the surviving patients all bottomed out and rose again (**Figure 1E**).

## Discussion

We reported the first study of plasma FXIII in patients with a larger sample size and multiple types of malignant solid tumors, investigating the association of plasma FXIII with the curative effects and prognosis of malignant solid tumors, as well as the influencing factors of plasma FXIII-A. We found for the first time that D2 = 1 mg/L was the cutoff point for the correlation between FXIII-A and D2 in patients with malignant solid tumors, and the interference of coagulation disorders on plasma FXIII-A was eliminated in D2 ≤ 1 mg/L, which provided a method to find a more realistic plasma FXIII-A level in patients with malignant solid tumors. We also found for the first time that plasma FXIII-A levels were abnormally significantly increased (FXIII-A >150%) in post-therapy patients, especially in NSCLC and lung metastasis patients; and the incidence of FXIII-A > 150% in lung adenocarcinoma was 16 times higher than that in LUSC. FXIII-A > 150% proved to be an independent risk factor for disease progression in NSCLC patients (OR=5.74, 95% CI: 1.20-27.60, *p* = 0.029), predicting poor efficacy. The marked decrease in plasma FXIII-A (FXIII-A < 40%) was related to serious complications such as coagulation disorders and poor prognosis with short survival (median survival time was 4 months).

These results help to clarify the contradictory results of the reported studies. Fluctuating consumption of plasma FXIII caused by sudden coagulation disorders (bleeding, hyperfibrinolysis and acute thrombosis) is a factor that must be considered in the analysis of plasma FXIII in patients with malignant tumors. In the present study, we found that the plasma FXIII-A level was significantly negatively correlated with D2 and FDP in D2 > 1 mg/L but uncorrelated with D2 or FDP in D2 ≤ 1 mg/L, suggesting that coagulation and fibrinolysis maintain a dynamic balance and plasma FXIII-A in patients with malignant solid tumors were free from coagulation disorders when D2 ≤ 1 mg/L. Presently, the reference range for D2 is 0-0.5 mg/L. The average D2 level in a variety of cancer patients is higher than 0.5 mg/L and close to 1 mg/L (19). Fijalkowska et al. showed that the median concentration of D2 in patients with lung cancer was 0.84 mg/L (20). Obviously, the high risk of thrombosis in cancer patients may be overestimated according to the current D2 reference range, and it does not universally apply to cancer patients. This study suggested that D2 = 1 mg/L was not only a reasonable inflection point for the correlation between FXIII-A and D2 but also the cutoff point of coagulation disorder in cancer patients, which is of great significance for the redefinition of D2 reference range and the judgment of coagulation disorders in cancer patients.

Previous studies mainly found a decrease in plasma FXIII, while few studies showed an increase in plasma FXIII, and the level of plasma FXIII-A was only relatively elevated but not out of the normal range. We found for the first time an abnormally significant increase in plasma FXIII-A in post-therapy patients with malignant solid tumors, especially in patients with lung adenocarcinoma and lung metastases. What is the cell source of plasma FXIII-A in the patients with malignant solid tumors? Why is the abnormal increase in plasma FXIII-A associated with treatment and lung cancer tissue specificity? We conducted the following preliminary exploration and speculation on these issues.

The origin of plasma FXIII-A has been a debate for a long period of time. Cell populations of bone marrow origin have long been considered the major source of plasma FXIII-A, and platelets are the most important cell source (21–23). Platelets harbor remarkably high concentrations of FXIII-A within their cytoplasm, with a single platelet accruing 60 ± 10 fg, corresponding to 3% of total platelet protein (24). However, studies have shown that the decrease in plasma FXIII-A is lower than expected in the patients with myelosuppression or thrombocytopenia (22, 23, 25), suggesting that there are some other cell populations responsible for the synthesis of FXIII-A in the case of highly impaired FXIII-A production by bone marrow cells. Kiss, F et al. reported that the expression of FXIII-A in B cell acute lymphoblastic leukemia cells is significantly increased (8). Resident macrophages were found to be the cell source of plasma FXIII-A in tissue-specific knockout mice (26). Porrello A et al. found that the transcription level of FXIII-A increased 2.5-fold in monocytes in the spleen of a mouse model of LUSC (13). Our results showed that plasma FXIII-A levels were significantly positively correlated with the numbers of monocytes and lymphocytes and related to platelets to some extent, which was consistent with these reports. However, there was no abnormal increase or decrease in blood cell count when FXIII>150%. Moreover, previous studies have shown that plasma FXIII-A decreased during inflammation (27, 28), and we found that the leukocyte count was not associated with plasma FXIII-A, and in the normal range when FXIII>150% in the present study. The results suggested that the abnormal increase in plasma FXIII-A was not associated with the number of circulating blood cells, treatment-related inflammation or cell damage caused by antitumor therapy but was more likely associated with the increased expression and release of certain cells induced by malignant tumors.

Remarkably, resident macrophages were found to be the cell source of plasma FXIII-A in tissue-specific knockout mice (26). Porrello A et al. found that the dense infiltration of TMAs with high FXIII-A expression in LUSC was correlated with poor survival (13). TAMs participate in the regulation of tumor development by polarization: M1 TMAs inhibit tumors; M2 TMAs promote tumors. FXIII-A mRNA and protein expression was significantly upregulated in M2 macrophages, and FXIII-A could be a marker for M2 macrophages (29). Moreover, FXIII-A cannot be secreted through the classical pathway due to the lack of a secreted signal peptide, and the secretory pathway by which FXIII-A is released from the cells also remains unknown. However, FXIII-A in macrophages was reportedly exogenous to the cell surface *via* Golgi vesicles in response to stimulation (25). It seems that M2 macrophages are likely to be one of the main cell sources of abnormally elevated plasma FXIII-A in patients with malignant solid tumors.

M2 TAMs are associated with immunosuppression, resistance to chemotherapy drugs, and progression and migration of malignant tumors (30, 31). Tumor cells construct tumor-promoting immune microenvironment by inducing M2 TMAs polarization. The association between abnormal elevation of plasma FXIII-A and polarization of M2 TMAs and the role of FXIII-A mediated polarization of TMAs in malignant tumors deserve our further exploration.

**Figure 4:**
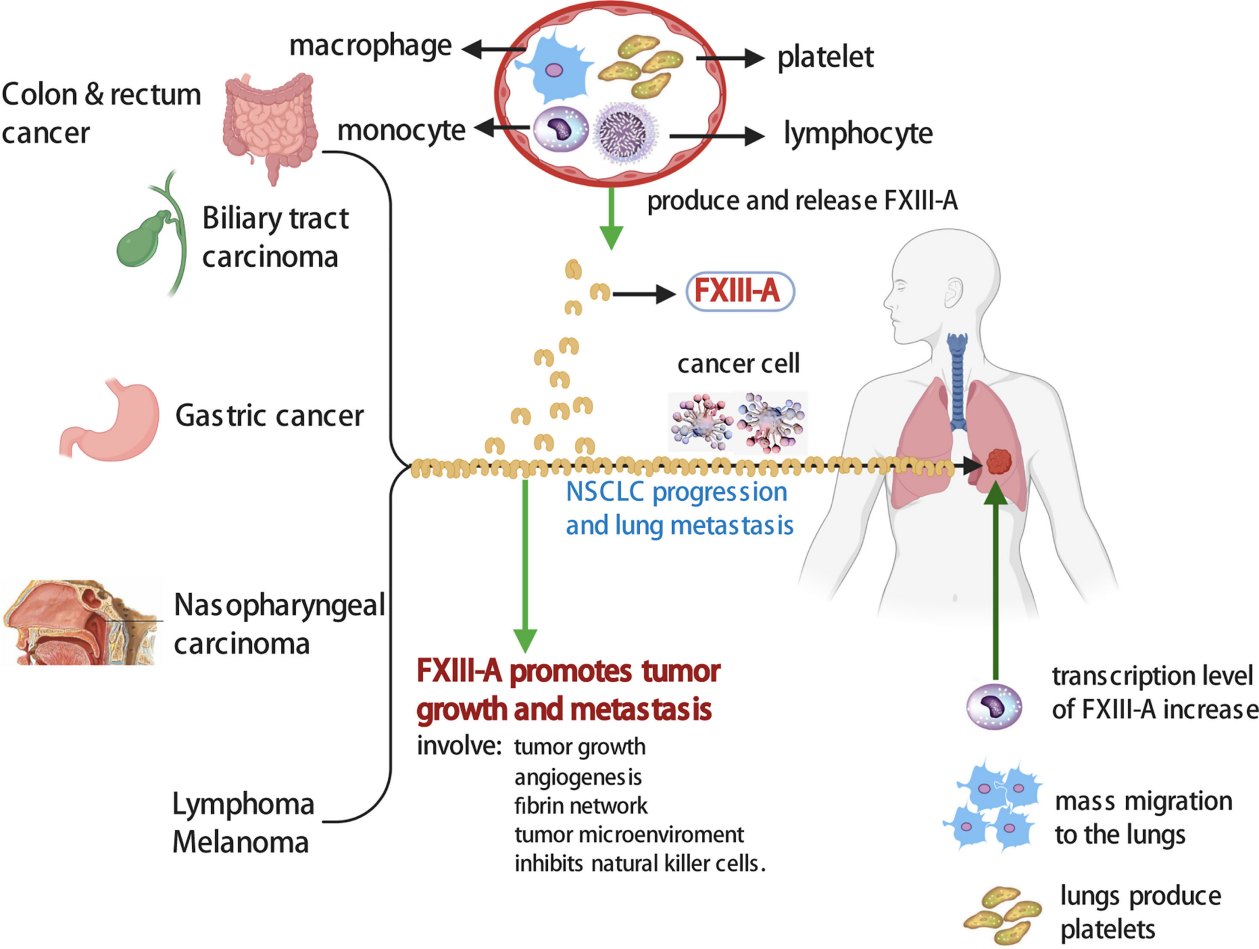
The increase of FXIII-A level promotes NSCLC progression and lung metastasis. Plasma FXIII-A level in some post-therapy NSCLC and lung metastasis patients were significant increase. Lung is an organ with considerable hematopoietic potential, produce numbers of platelets. Platelets, macrophages, monocytes and lymphocytes might be the sources of plasma FXIII-A in cancer patients, and M2 TAMs might be responsible for the abnormal increase of plasma FXIII-A. The expression of FXIII-A is significantly upregulated in M2 TAMs. Numbers of macrophages migrate to the lung in cancer patients. The increase of FXIII-A level promotes growth and metastasis of malignant tumor.

Interestingly, Lefrancais et al. found that the lung was an organ with considerable hematopoietic potential and produces more than half of the total platelet count in a mouse model (32). Moreover, some chemotherapeutic drugs, such as paclitaxel and doxorubicin, promote the expression of chemokines in lung tissue to attract mononuclear macrophages and promote lung metastasis of cancer cells (33, 34). Researchers believe that the role of chemotherapy in promoting metastasis may be widespread. We speculate that the increase in plasma FXIII-A may be derived partially from cells in pulmonary, as the treatment measures or the progression of tumors stimulate pulmonary hematopoiesis (**Figure 4**). This study showed that the incidence of FXIII-A > 150% in lung adenocarcinoma was much higher than that in LUSC, which was consistent with recently reported research showing that adenocarcinoma was more prone to hematogenous and lymph node metastasis than squamous carcinoma (35). Moreover, in addition to the possible specific association between lung adenocarcinoma and FXIII-A, the possible influence of chemotherapy types and drugs should also be considered, which will be an interesting research point in subsequent studies.

Our results showed that plasma FXIII-A levels were persistently increased in NSCLC patients with disease progression and significantly positively correlated with CEA levels. We confirmed that FXIII-A > 150% was an independent risk factor for the disease progression in NSCLC. Existing studies suggested that FXIII-A may promotes tumor growth and metastasis in the following ways (**Figure 4**): (1) FXIII-A is involved in the stabilization of the fibrin network within and around neoplastic tissues, which facilitates tumor-matrix generation, tumor angiogenesis and metastasis (14, 36). (2) FXIII-A is not only involved in the proliferation and migration of vascular endothelial cells and inhibits their apoptosis but also exerts its proangiogenic effect by downregulating the expression of thrombospondin-1 (37). The role of FXIII-A in angiogenesis has been verified in a variety of animal models (38, 39). (3) FXIII-A is involved in the immune escape of cancer cells. Palumbo et al. showed that FXIII was a significant determinant of metastatic potential and supports metastasis by impeding NK cell-mediated clearance of tumor cells (16). Presently, the mechanism of FXIII in cancer is still speculative and needs further exploration.

We found that patients with FXIII-A < 40% had a short survival time and were complicated with serious effusion and coagulation disorders. Studies have shown a marked decrease in plasma FXIII-A due to its heavy consumption during acute thrombosis and hyperfibrinolysis (40–42). Plasma FXIII levels in the lowest quartile 24 h post-lysis proved to be an independent predictor of mortality by 14 days post-event (43). Plasma FXIII-A in patients with acute ischemic stroke decreased significantly from day 0 to day 1, which was associated with an unfavorable short-term clinical outcome (44). Our results agree with these studies, suggesting a significant decrease in plasma FXIII-A due to acute consumption of FXIII-A, which is associated with coagulation disorders and short-term death in patients. Cancer patients often have aberrant blood coagulation mechanisms and are at significantly increased risk for arterial and venous thromboembolism, especially those receiving systemic chemotherapy (45–48). Moreover, some cancer patients have relatively insidious clinical manifestations, such as an asymptomatic hypercoagulation state and thromboembolism. D2 is not specific for the detection of thrombosis, especially in cancer patients. Plasma FXIII-A could enhance the sensitivity of monitoring coagulation disorders. Immunohistological investigations demonstrated the deposition of FXIII at interfaces of adjacent endothelial cells and between the cells and the filter support. The mechanism of the effect of FXIII on endothelial cell permeability has not been revealed, but a few clinical and experimental animal studies have demonstrated that FXIII decreases the enhanced vascular permeability induced by various stimuli, and plasma FXIII levels are negatively correlated with fluid loss (49–51). In summary, the sharp decrease in plasma FXIII-A (FXIII-A < 40%) indicates a very poor prognosis and coagulation disorders. Other possible causes of FXIII-A decline remain to be studied.

Our study has some limitations. First, although the total sample size was larger, the samples of some types of malignant tumors were small; therefore, the specific association between the abnormal elevation of plasma FXIII-A and lung malignancy still needs to be confirmed with a larger sample size and more types of malignant solid tumors. Second, the number of NSCLC patients who started monitoring before treatment was small; thus, we chose a relatively consistent monitoring period.

In conclusion, we found the optimal cutoff value of D2 to clarify the ambiguity surrounding the changes in FXIII-A caused by coagulation disorders and confirmed that plasma FXIII-A has the potential as a real-time monitoring biomarker with bidirectional indicator effects to simultaneously monitor coagulation disorder, curative effects and prognosis in patients with malignant solid tumors. The significant increase in plasma FXIII-A (FXIII-A > 150%) was associated with the progression of NSCLC, predicting poor efficacy. The marked decrease in plasma FXIII-A (FXIII-A < 40%) was related to serious complications such as coagulation disorders, as well as a poor prognosis with short survival time.

## Data Availability Statement

The raw data supporting the conclusions of this article will be made available by the authors, without undue reservation.

## Ethics Statement

The studies involving human participants were reviewed and approved by the institutional Ethics Committee of The Second XiangYa Hospital. Written informed consent for participation was not required for this study in accordance with the national legislation and the institutional requirements.

## Author Contributions

YL and GZ conceptualized and designed the manuscript. YL, BL and GZ developed the methodology. YL, GZ, CH, ZZ, BL, JL, YZ, MD and WY acquired the data. YL, GZ, BL, JL, ZZ and YZ analyzed and interpreted the data. YL, GZ, BL, and ZZ wrote, reviewed, and/or revised the manuscript. All authors contributed to the article and approved the submitted version.

## Funding

The research was funded by the National Natural Science Foundation of China (Grant Nos. 81700168 and 81900170).

## Conflict of Interest

The authors declare that the research was conducted in the absence of any commercial or financial relationships that could be construed as a potential conflict of interest.

## Publisher’s Note

All claims expressed in this article are solely those of the authors and do not necessarily represent those of their affiliated organizations, or those of the publisher, the editors and the reviewers. Any product that may be evaluated in this article, or claim that may be made by its manufacturer, is not guaranteed or endorsed by the publisher.

## Abbreviations

APTT, activated partial thromboplastin time; CEA, carcino-embryonic antigen; D2, D-dimer; FDP, fibrin degradation products; FXIII, coagulation factor XIII; FXIII-A, coagulation factor XIII subunit A; NSCLC, non-small cell lung carcinoma; PT, prothrombin time; TAMs, tumor-associated macrophages; TME, tumor microenvironment.
